# Sleep and Anabolic/Catabolic Hormonal Profile in Sedentary Middle-Aged Adults: The FIT-AGEING Study

**DOI:** 10.3390/ijms232314709

**Published:** 2022-11-25

**Authors:** Sol Mochón-Benguigui, Almudena Carneiro-Barrera, Manuel Dote-Montero, Manuel J. Castillo, Francisco J. Amaro-Gahete

**Affiliations:** 1 EFFECTS-262 Research Group, Department of Medical Physiology, School of Medicine, University of Granada, 18016 Granada, Spain; 2 Department of Psychology, Universidad Loyola Andalucía, 41704 Seville, Spain; 3 Sleep and Health Promotion Laboratory, Mind, Brain and Behavior Research Centre, University of Granada, 18011 Granada, Spain; 4 PROmoting FITness and Health through Physical Activity Research Group (PROFITH), Sport and Health University Research Institute (iMUDS), Department of Physical Education and Sports, Faculty of Sport Sciences, University of Granada, 18071 Granada, Spain; 5 Centro de Investigación Biomédica en Red Fisiopatología de la Obesidad y Nutrición (CIBERobn), Instituto de Salud Carlos III, 28029 Madrid, Spain

**Keywords:** hormone, dehydroepiandrosterone sulphate, testosterone, somatotropin, cortisol, actigraphy

## Abstract

Sleep quality plays an important role in the modulation of several aging markers. This influence could be explained by aging-induced hormonal changes. Indeed, poor sleep quality has been associated with the development of several endocrine-related health complications. This study examined the relationship of both subjective and objective sleep quantity and quality, with basal levels of selected plasma anabolic and catabolic hormones in sedentary middle-aged adults. A total of 74 volunteers (52.7% women; aged 53.7 ± 5.1) were recruited for this study. Subjective sleep quality was assessed by the Pittsburgh Sleep Quality Index (PSQI; higher scores indicate worse sleep quality), and objective sleep quality parameters (total sleep time [TST], wake after sleep onset [WASO], and sleep efficiency [SE]) were measured using a wrist-worn accelerometer. Basal levels of plasma dehydroepiandrosterone sulphate (DHEAS), total testosterone, sex hormone binding globulin (SHBG), somatotropin, and cortisol levels, were determined. Free testosterone was calculated from the total testosterone and SHBG levels. No associations of global PSQI score, TST, WASO, and SE with DHEAS, free testosterone, and somatotropin plasma levels were found, neither in men nor in women (all *p* ≥ 0.05). Global PSQI score was inversely related to cortisol plasma levels in women (*p* = 0.043). WASO was positively associated with cortisol plasma levels, while SE was negatively associated with cortisol plasma levels in women (all *p* ≤ 0.027). Sleep quality is not related to levels of plasma anabolic hormones, but to levels of catabolic hormones, in sedentary middle-aged adults. Therefore, these results suggest that potential changes in aging biomarkers associated with sleep disturbances, could be mediated by age-related changes in the catabolic endocrine system.

## 1. Introduction

Aging comes with changes in hormonal secretion, which in turn results in subsequent increases/decreases in their circulating levels [1]. Among hormones, those better characterizing these age-related changes may be somatotropin and testosterone (anabolic hormones), which decrease with age [1,2,3,4], and cortisol, an important catabolic hormone that increases during the aging process [1,2,5]. In addition to cortisol, another product of the adrenal cortex secretory activity is dehydroepiandrosterone (DHEA). This hormone—which mainly circulates as a sulphate ester (dehydroepiandrosterone sulphate [DHEAS]) [6], and whose physiological action remains partially uncertain [2]—is the most abundant corticosteroid in circulation [7], being one of the blood parameters that consistently decreases the most over the years [1,2,8]. Importantly, the global prevalence of testosterone deficiency ranges from 10 to 40%, being especially important in men aged 45–50 years [9]. Moreover, a gradual decline of testosterone plasma levels is observed in women until menopause, with a sudden decrement of 15–22% after this biological stage [10]. DHEA levels also suffer a gradual decrease until menopause (60%) [11]. In this sense, the importance of early detection and timely treatment of these alterations has been recognized [12], and the eventual exogenous administration of somatotropin, testosterone, and DHEA as a true anti-aging therapy, has been highlighted [13]. However, changes in lifestyle regulating the hormonal status could be considered, before the application of the above-mentioned treatments [14].

Previous studies have found that sleep quality plays an important role in the modulation of several aging markers (i.e., body composition [15,16,17], energy metabolism [18,19], cardiometabolic risk [20,21], immune function and hemostasis [22,23,24], S-Klotho anti-aging protein [25,26], and physical activity and fitness [27,28,29], among others). This influence could be explained by aging-induced hormonal changes [30]. Indeed, it has been suggested that poor sleep quality is associated with the development of several endocrine-related health complications (i.e., obesity, diabetes, metabolic syndrome, and reduced fecundity) [31,32,33], increasing all-cause morbidity and mortality risk [34]. Interesting findings evidencing the relationship of sleep quantity and quality, with DHEAS, testosterone, somatotropin, and cortisol levels, have been reported by diverse authors [35,36,37,38,39,40,41,42]. However, there is controversy in this field of knowledge, since some studies found no association between sleep and the levels of the previously mentioned hormones [43,44,45,46,47]. These discrepancies may be explained by age differences of the subjects included, different protocols and methodologies used to assess sleep quantity and quality, and timing of the determination of hormones. Our research group has previously shown that a poor subjective sleep quality is associated with an altered body composition status (i.e., decreased bone mineral density and lean mass, and increased fat mass) [48], energy metabolism (i.e., lower basal fat oxidation) [49], cardiometabolic risk profile (i.e., worse plasma lipid profile), hematological parameters (i.e., impaired hemostasis), S-Klotho anti-aging protein levels (i.e., decreased S-Klotho plasma levels) [50], and physical activity and fitness (i.e., lower levels of overall physical activity, maximal oxygen uptake [VO_2_max] and muscular strength) [51], all of them widely considered as aging biomarkers. It is therefore of scientific and clinical interest to investigate whether poor sleep quality is also associated with age-induced changes in the endocrine system.

Hence, this study was aimed at evaluating the association of both subjective (i.e., global Pittsburgh Sleep Quality Index [PSQI] score) and objective (i.e., total sleep time [TST], wake after sleep onset [WASO], and sleep efficiency [SE]) sleep quantity and quality, with basal levels of plasma DHEAS, testosterone, somatotropin, and cortisol, in sedentary middle-aged adults. Considering the key role of the endocrine system in the regulation of human physiological processes, and sleep as a major modulator of hormonal release, we hypothesized that individuals with an unhealthy sleep pattern would also present with alterations in plasma hormone levels.

## 2. Results

Descriptive characteristics of study participants by sex, are shown in Table 1. Significant differences in height, weight, body mass index (BMI), lean mass index (LMI), DHEAS, free testosterone, total testosterone, sex hormone binding globulin (SHBG), somatotropin, and TST were observed between men and women (all *p* ≤ 0.006). A poor subjective sleep quality (global PSQI score > 5) was identified in 40.3% of our cohort.

**Figure 1:**
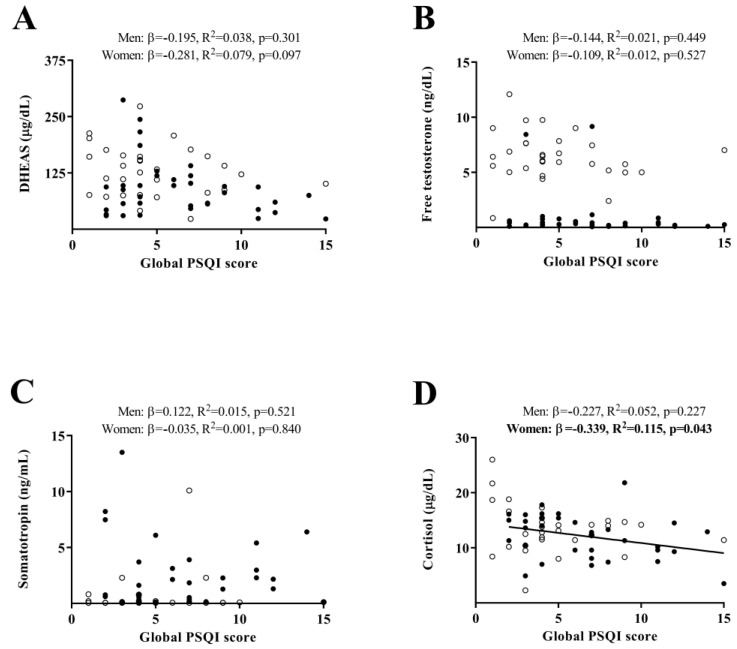
Association of subjective sleep quantity and quality with levels of plasma hormones (Panel (**A**): DHEAS, Panel (**B**): Free testosterone, Panel (**C**): Somatotropin, and Panel (**D**): Cortisol) in sedentary middle-aged adults. *β* (standardized regression coefficient), *R*^2^, and *p*-value from a simple linear regression analysis. Significant *p*-values (<0.05) are in bold. Open circles represent men, closed circles represent women, and the straight solid line represents women. PSQI: Pittsburgh Sleep Quality Index; DHEAS: dehydroepiandrosterone sulphate.

Figure 1 shows the association of subjective sleep quantity and quality, with levels of plasma hormones. No significant associations were found between global PSQI score and DHEAS, free testosterone, or somatotropin plasma levels, neither in men nor in women (all *p* ≥ 0.05, Figure 1A–C). We found a negative association of global PSQI score with cortisol plasma levels in sedentary middle-aged women (*β* = –0.339, *R*^2^ = 0.115, *p* = 0.043, Figure 1D), while no association of global PSQI score with cortisol was found in men (*p* ≥ 0.05, Figure 1D).

**Figure 2:**
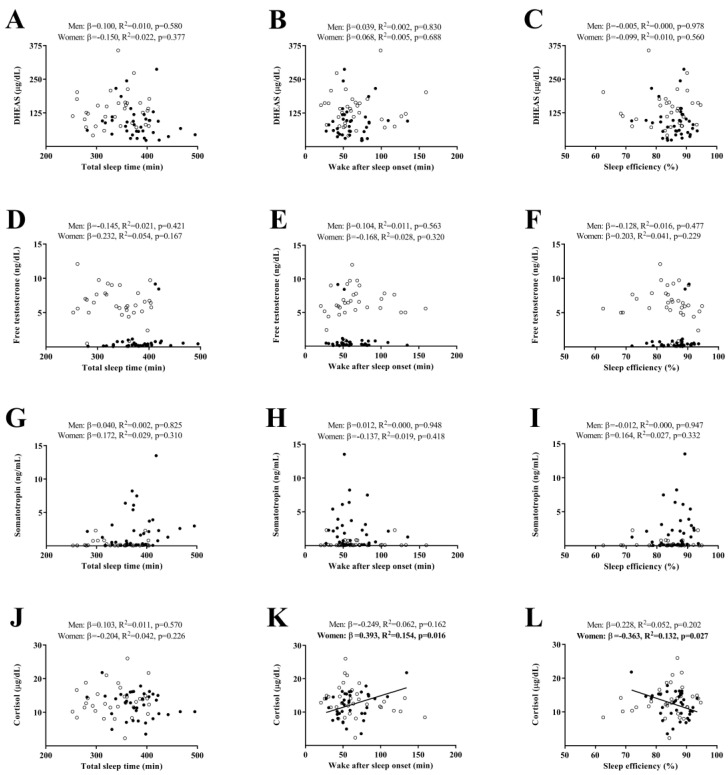
Association of objective sleep quantity and quality with levels of plasma hormones (Panel (**A**–**C**): DHEAS, Panel (**D**–**F**): Free testosterone, Panel (**G**–**I**): Somatotropin, and Panel (**J**–**L**): Cortisol) in sedentary middle-aged adults. *β* (standardized regression coefficient), *R*^2^, and *p*-value from a simple linear regression analysis. Significant *p*-values (<0.05) are in bold. Open circles represent men, closed circles represent women, and the straight solid line represents women. DHEAS: dehydroepiandrosterone sulphate.

Figure 2 shows the association of objective sleep quantity and quality, with levels of plasma hormones. No associations of TST, WASO, and SE with DHEAS, free testosterone, and somatotropin plasma levels were found, neither in men nor in women (all *p* ≥ 0.05, Figure 2A–I). No association of TST with cortisol plasma levels was found, neither in men nor in women (all *p* ≥ 0.05, Figure 2J). WASO was positively associated with cortisol plasma levels, while a significant negative association was found between SE and cortisol plasma levels in women (*β* = 0.393, *R*^2^ = 0.154, *p* = 0.016 and *β* = –0.363, *R*^2^ = 0.132, *p* = 0.027, respectively, Figure 2K,L). No association of WASO or SE with cortisol was found in men (all *p* ≥ 0.05, Figure 2K,L).

All of the above-mentioned findings persisted after controlling for age, fat mass index (FMI), and LMI (all *p* ≥ 0.05, Supplementary Material Table S1). In addition, no significant associations of either subjective or objective sleep quantity and quality—with total testosterone, SHBG, and DHEAS/cortisol, free testosterone/cortisol, total testosterone/cortisol, and somatotropin/cortisol ratios—were found (all *p* ≥ 0.05, Supplementary Material Table S2).

## 3. Discussion

This study was aimed at investigating the potential associations between sleep quantity and quality, with levels of plasma hormones that change with age, such as DHEAS, free testosterone, somatotropin, and cortisol, in sedentary middle-aged adults. According to our results, no associations of sleep with levels of plasma anabolic hormones, were found in our study. However, worse perceived sleep quality was associated with lower levels of cortisol in sedentary middle-aged women. Interestingly, WASO was positively associated with cortisol plasma levels, while a significant negative association was found between SE and cortisol plasma levels in sedentary middle-aged women. Thus, potential alterations during the aging process induced by sleep disturbances, could be mediated by age-related changes in the catabolic endocrine system.

Sleep has been postulated as a major modulator of hormonal release [33]. Variability between days, in duration of sleep and sleep schedules, may therefore promote a mismatch between behavioral cycles and innate circadian rhythms, leading to a dysregulation of metabolic and endocrine functions [20]. Sleep plays an important role in testosterone secretion, since it is mainly released during sleep time in young adult men [52]. Concretely, testosterone plasma levels rise upon falling asleep, peak at the phase of first rapid eye movement, and remain at the same levels until awakening [52]. Thus, sleep fragmentation could be associated with reduced testosterone plasma levels. Similarly, DHEA plasma levels are regulated according to a circadian rhythm [8]. Uncertain findings have been reported regarding the relationship between sleep, and testosterone or DHEAS. In our study, we did not find any significant association between sleep quantity and quality and both free testosterone and DHEAS, neither in men nor in women. In accordance with our results, Mohammadi et al. [43,44] did not observe significant differences in testosterone and DHEA plasma levels between (i) two insomnia subgroups (i.e., paradoxical vs. psychophysiological insomnia) vs. normal sleepers, and between (ii) three subtypes of obstructive sleep apnea vs. normal sleepers. Moreover, our results are also in line with another cross-sectional study that investigated the association of sleep duration and architecture, with testosterone in healthy men [45]. In addition, Ruge et al. [46] found no association between self-reported sleep quality and serum levels of male reproductive hormones, in men. Regarding sleep duration, they found a statistically significant association from continuous analysis of sleep duration and hormones [46]. However, no association between restrictive sleep and hormones was noted in this work, obtaining a significant relationship between excessive sleep and reduced concentrations of testosterone in the categorized analyses [46].

In contrast, Barrett-Connor et al. [35] showed that lower testosterone levels were associated with decreased SE and increased WASO in a cohort of 1312 older men, suggesting that this association could be largely explained by adiposity. Similarly, a review by Burschtin et al. [37] concluded that serum testosterone is lower in men with obstructive sleep apnea compared with healthy subjects, proposing that these differences could be explained by alterations of sleep architecture, periods of low oxygen saturation, and changes in control hormone levels. These inconsistencies could be potentially explained by methodological considerations regarding the assessment of sleep quantity and quality, sample size, and participant’s age and biological conditions [46]. Thereby, future studies with greater sample sizes, recruiting participants without testosterone or sleep disturbances, including objective and reliable measurement tools, and evaluating possible covariates as body composition, are certainly needed.

Sleep also plays a key role in the somatotropin release since its most significant production occurs during deep or slow-wave sleep in healthy adults [53]. Sleep pattern alterations (i.e., insomnia, or sleep-disordered breathing, among others) have been positioned as potential disruptors of somatotropin synthesis and secretion [54]. In our study, we did not find any significant relationship of sleep quantity and quality with somatotropin plasma levels, neither in men nor in women. These results did not concur with those obtained by Redwine et al. [38], who compared the effects of both nocturnal sleep and partial night sleep deprivation, on the secretory profile of somatotropin in healthy male volunteers, observing that experimental induction of sleep loss resulted in a delay in the nocturnal elevation of somatotropin. Moreover, a study by Brandenberger et al. [39] compared 24 h rhythms of somatotropin in both healthy day-active males and night workers, concluding that there was no significant difference in the total amount of secreted somatotropin during the 24 h, between them. Interestingly, they observed a lower sleep-related somatotropin pulse in night workers, which was subsequently compensated for by large individual pulses occurring during waking periods. Therefore, it seems that although a compensatory mechanism is present when sleep and circadian rhythms are misaligned [39,55], the long-term metabolic consequences induced by sleep-related changes, on somatotropin pulses released throughout the 24 h, are not clearly established.

Sleep also plays a significant role in cortisol release, as the hypothalamic–pituitary–adrenal axis is acutely inhibited during early slow-wave sleep [53]. Disturbances of sleep homeostasis are usually related to a rise in hypothalamic–pituitary–adrenal axis activity, causing an increase in cortisol [56]. However, the majority of studies investigating the relationship between sleep and cortisol release, are based on sleep-deprivation protocols—characterized by inducing stress per se—which cannot be extrapolated to a real context [56]. In the current study, we found that a higher global PSQI score (i.e., worse perceived sleep quality) was related to lower cortisol plasma levels in women, while no relationship was noted in men. These findings are not in accordance with those reported by Asarnow [41], who suggested that both experimentally induced and naturalistically observed poor sleep affects cortisol reactivity and recovery, following an acute exposure to a stressor agent in healthy adults. Moreover, van Dalfsen et al. [42] suggested that reduced subjective sleep quality might potentiate the reactivity of the hypothalamic–pituitary–adrenal axis, through physical and psychosocial stressors. They also concluded that objective diminished SE and/or increased awakenings through the night may promote cortisol responsiveness, particularly by psychosocial stress [42]. Nevertheless, Bani-Issa et al. [47] showed that morning and bedtime cortisol levels were not significantly correlated with self-reported sleep quality among healthy healthcare women, which also is not in accordance with our findings.

As previously mentioned, investigating the impact of sleep on stress reactivity of the hypothalamic–pituitary–adrenal axis depends on several methodological issues including stressor type, and seasonal stage or age, among others [42,57]. This fact could therefore be a potential explanation for the above-mentioned uncertain results. Interestingly, Van Cauter et al. [53] pointed out that aging is associated with a significant increment of evening cortisol levels. In this sense, damaging effects seem to be more marked at the time of the trough of the rhythm, than at the time of the peak. In this sense, one major limitation is that cortisol is usually measured at the end of the sleep-deprivation procedure [56]. Therefore, further studies measuring both morning and evening cortisol levels, or the entire 24-h profile using saliva samples would be desirable to robustly establish the link between these variables.

The methodology used in the sleep parameters measurement could also contribute to this discordance. Concretely, our study showed differences between subjective and objective sleep assessments, since WASO was positively associated with cortisol plasma levels, and SE was negatively related to cortisol plasma levels in women. Previous studies have revealed weak or inconsistent correlations of subjective (i.e., PSQI scores) with objective (e.g., actigraphy and polysomnography) measures [58,59,60]. In fact, it has been shown that the PSQI and the accelerometer may assess different attributes of sleep [61]. Given this limitation, using both complementary measurement methods to obtain detailed information, is suggested [62]. PSQI may reflect the general psychological state of the person, rather than real quantity or quality of sleep [59]. Furthermore, it is still not well-defined what a “good night’s sleep” truly involves in the sleeper’s perception, since many factors can contribute to general sleep quality [63].

Robust clinical and research implications can be found in our study, which provides evidence of the relationship of sleep with cortisol plasma levels, in sedentary middle-aged women. Considering the importance of early detection and timely treatment of aging-induced hormonal alterations, changes in lifestyle (i.e., sleep behaviors, among others) regulating the hormonal status have to be considered, in order to avoid the development of several endocrine-related health complications. However, several limitations should be acknowledged and addressed in further studies. The main limitation of the present work was the cross-sectional study design, which does not allow determination of cause–effect relationships. Therefore, well-designed intervention studies are needed to robustly establish causal relationships. In addition, the use of actigraphy may under/over-estimate sleep parameters since it is not the gold-standard method to assess sleep quantity and quality. Thus, future research should include polysomnography (i.e., the gold standard method) to appropriately analyze not only sleep duration and efficiency, but also other significant sleep outcomes including sleep architecture (i.e., rapid eye movement [REM] sleep stage, and non-REM sleep stages [N1, N2, and N3]). Moreover, levels of plasma hormones were measured at a single time point instead of frequent sampling. Therefore, future studies should implement the collection of several samples throughout the day, in order to understand the behavior of the studied parameters over 24 h. Finally, our study sample only included sedentary middle-aged adults, so these findings cannot be generalized to other populations. Thus, evaluation of different populations is necessary.

## 4. Materials and Methods

### 4.1. Study Protocol and Participants

The present cross-sectional study was conducted in the framework of the FIT-AGEING project [64], an exercise-based randomized controlled trial (clinicaltrial.gov: ID: NCT03334357). The Human Research Ethics Committee of the Regional Government of Andalucía approved the rationale, design, and methodology of the study [0838-N-2017], complying with the ethical principles described in the last revised Declaration of Helsinki. A detailed explanation of the study methodology can be found elsewhere [64]. Eligible participants were adults aged 40–65 years, with a BMI between 18.5 and 35 Kg/m^2^, stable weight (less weight change than 3 kg over the last 3 months), and non-physically active (less than 20 min of vigorous-intensity physical activity or less than 3 days a week). Participants with a diagnosis of any physical or psychological disease, or under medical treatment, as well as being pregnant, were excluded from the study. A total of 74 sedentary healthy middle-aged volunteers (52.7% women, 53.7 ± 5.1 years old, 26.7 ± 3.8 kg/m^2^) were enrolled in the study. Upon meeting the inclusion criteria, participants received a full explanation of the study aims and procedures, signed an informed consent form, and underwent a complete history and physical examination prior to their participation. All assessments were performed at the Sport and Health University Research Institute (iMUDS) (Granada, southern Spain) during the months of September and October in 2016 and 2017.

### 4.2. Measurements

#### 4.2.1. Anthropometry and Body Composition

A pre-validated electronic scale and stadiometer (model 799, Electronic Column Scale, Hamburg, Germany) were used to measure body weight and height. BMI was calculated as $Body\;weight\;\left(\mathrm{kg}\right)⁄Height^{2}\;\left(m^{2}\right)$ [65].

A dual-energy X-ray absorptiometry scanner (Hologic, Inc., Bedford, MA, USA) was used to determine fat mass and lean mass. FMI and LMI were calculated as: $Fat\;mass\;\left(\mathrm{kg}\right)⁄{Height}^{2}\;\left(m^{2}\right),$ and $Lean\;mass\;\left(\mathrm{kg}\right)\;/Height^{2}\;\left(m^{2}\right)$, respectively.

#### 4.2.2. Sleep Quantity and Quality

Subjective sleep quantity and quality were assessed using the PSQI scale [66], a 19-item scale that provides 7 component scores (ranges 0–3): (i) subjective sleep quality, (ii) sleep latency, (iii) sleep duration, (iv) habitual sleep efficiency, (v) sleep disturbances, (vi) use of sleeping medication, and (vii) daytime dysfunction. The sum of component scores was used to compute the global PSQI score, which ranged from 0 to 21. Lower global scores denote a healthier sleep quality, whereas global scores greater than 5 are associated with poor sleep quality.

Objective sleep quantity and quality were determined using a wrist-worn accelerometer (ActiGraph GT3X+, Pensacola, FL, USA) during 7 consecutive days (24 h/day) [64]. Participants received detailed information regarding how to wear the accelerometer (on the non-dominant wrist) and were also asked to remove it only during water activities such as swimming or bathing. A 7 day sleep diary was provided to record bedtime, wake up time, and the time they removed the device each day. The accelerometers used an epoch length of 1 s and a frequency rate of 100 Hz to store raw accelerations [67]. The raw accelerations were exported and converted to the “.csv” format using ActiLife software (version 6.13.3, ActiGraph, Pensacola, FL, USA). The raw “.csv” files were then processed with GGIR package (version 1.5-12, https://cran.r-project.org/web/packages/GGIR/; accessed on 13 April 2022) in R (version 3.1.2, https://www.cran.r-project.org/https://cran.r-project.org/web/packages/GGIR/; accessed on 13 April 2022). Signal processing included: (i) autocalibration according to the local gravity [68], (ii) detection of sustained abnormal high accelerations, (iii) detection of the non-wear time, (iv) calculation of the Euclidean Norm Minus One (ENMO), (v) identification of waking and sleeping hours with an automatized algorithm [69], and (vi) imputation of detected non-wear time and abnormal high values. The variables obtained from actigraphy recordings were TST (total amount of time spent in bed minus sleep onset latency), WASO (the sum of wake times from sleep onset to the final awakening), and SE (percentage of sleep time over the bedtime) [70]. Only the participants wearing the accelerometers for ≥16 h/day during at least 4 of 7 possible days (including at least 1 weekend day), were included in the final analyses. 

#### 4.2.3. Blood Sampling

A 10 mL peripheral blood sample was taken from the antecubital vein applying standard techniques, after 12 h fasting. Blood samples were collected in the morning (8:30 a.m.–10:00 a.m.) using prechilled ethylene diamine tetra-acetic acid-containing tubes (Vacutainer SST, Becton Dickinson, Plymouth, UK) and immediately centrifuged at 4000 rpm at 4 °C for 7 min. Subsequently, they were processed in a controlled-temperature room (22 ± 0.5 °C). Basal levels of plasma DHEAS, total testosterone, SHBG, somatotropin, and cortisol were measured using Beckman Coulter DxI 800 immunoassay system (Beckman Coulter Inc., Brea, CA, USA) which uses chemiluminescent technology. Free testosterone (i.e., the portion not bound to either SHBG or albumin) was calculated from the total testosterone and SHBG using the mass action equations described by Vermeulen et al. [71]. The results were expressed in μg/dL, ng/dL, nmol/L, ng/mL, μg/dL, and ng/dL, respectively. All participants were requested to abstain from drugs and/or caffeine (24 h before), to eat a pre-established dinner (i.e., boiled rice, tomato sauce, and plain egg omelet) before sampling, and to avoid any physical activity of moderate (24 h before) and/or vigorous (48 h before) intensity.

### 4.3. Statistical Analysis

Descriptive parameters are expressed as mean and standard deviation. Data were checked for normality with the use of distribution plots (i.e., visual check of histograms, Q-Q plots, and box plots) and the Shapiro–Wilk test. Sex differences were determined using independent samples *t*-test. Given that significant interactions were observed between the sexes for plasma DHEAS, free testosterone, total testosterone, SHBG, and somatotropin, the analyses were performed separately for men and women.

Simple linear regression models were built to investigate the association of sleep quantity and quality (TST, WASO, SE, and global PSQI score) with levels of plasma hormones. Multiple linear regression models were also conducted in order to test these associations after adjusting for age, FMI, and LMI. Potential covariates were selected based on theoretical bases and statistical procedures (i.e., stepwise regressions). Both age and body composition are known to affect both sleep pattern and hormone secretion [1,72,73,74].

All analyses were performed using the Statistical Package for Social Sciences (SPSS, v. 23.0, IBM SPSS Statistics, IBM Corporation, Armonk, NY, USA) and graphical presentations were prepared using GraphPad Prism 6 (GraphPad Software, San Diego, CA, USA). *p* values less than 0.05 were considered statistically significant.

## 5. Conclusions

The current study indicates that sleep quantity and quality is not related to levels of plasma anabolic hormones (i.e., DHEAS, testosterone, and somatotropin) in sedentary middle-aged adults, but it is related to catabolic hormone levels (i.e., cortisol) in sedentary middle-aged women. Therefore, these results suggest that potential changes in aging biomarkers associated with sleep disturbances, could be mediated by age-related changes in the catabolic endocrine system.

## Figures and Tables

**Table 1:** Descriptive characteristics of participants.

	N	All		N	Men		N	Women	
Age (years)	74	53.66	(5.14)	35	54.39	(5.27)	39	53.01	(5.00)
*Anthropometry and body composition*									
Height (cm)	74	167.8	(9.81)	35	175.8	(6.48)	39	160.7	(6.10) *
Weight (kg)	74	75.73	(14.98)	35	87.38	(10.95)	39	65.28	(9.32) *
Body mass index (kg/m^2^)	74	26.72	(3.76)	35	28.32	(3.61)	39	25.27	(3.31) *
Fat mass index (kg/m^2^)	74	10.75	(3.13)	35	10.03	(3.23)	39	11.39	(2.93)
Lean mass index (kg/m^2^)	74	15.21	(2.88)	35	17.49	(2.02)	39	13.17	(1.80) *
*Hormones*									
DHEAS (μg/dL)	73	109.8	(66.95)	34	135.1	(66.45)	39	87.74	(59.87) *
Free testosterone (ng/dL)	73	3.40	(3.50)	34	6.37	(2.37)	39	0.81	(1.90) *
Total testosterone (ng/dL)	73	182.0	(182.6)	34	342.1	(128.8)	39	42.44	(76.66) *
SHBG (nmol/L)	73	47.02	(23.76)	34	35.79	(16.36)	39	56.81	(25.01) *
Somatotropin (ng/mL)	73	1.44	(2.54)	34	0.60	(1.77)	39	2.17	(2.88) *
Cortisol (μg/dL)	73	12.86	(4.18)	34	13.43	(4.49)	39	12.36	(3.87)
*Sleep quantity and quality*									
Global PSQI score	67	5.61	(3.47)	31	4.77	(3.15)	36	6.33	(3.62)
Total sleep time (min)	71	359.9	(48.85)	34	337.9	(46.30)	37	380.1	(42.44) *
Wake after sleep onset (min)	71	63.90	(27.44)	34	65.80	(32.45)	37	62.15	(22.19)
Sleep efficiency (%)	71	85.01	(6.29)	34	83.88	(7.53)	37	86.06	(4.75)

Data are presented as mean (standard deviation). * Significant differences between sexes obtained from an independent samples *t*-test (*p* < 0.05). DHEAS: dehydroepiandrosterone sulphate; SHBG: sex hormone binding globulin; PSQI: Pittsburgh Sleep Quality Index.

## Data Availability

All relevant data are included in the manuscript, Supplementary Materials, or will be made available upon request to the corresponding author.

## Supplementary Materials

The following supporting information can be downloaded at: https://www.mdpi.com/article/10.3390/ijms232314709/s1, Table S1: Association of sleep quantity and quality with dehydroepiandrosterone sulphate, free testosterone, somatotropin, and cortisol (Model 0) adjusted by age (Model 1), by fat mass index (Model 2), and by lean mass index (Model 3); Table S2: Association of sleep quantity and quality with total testosterone, sex hormone binding globulin, and dehydroepiandrosterone sulphate/cortisol, free testosterone/cortisol, total testosterone/cortisol, and somatotropin/cortisol ratios (Model 0) adjusted by age (Model 1), by fat mass index (Model 2), and by lean mass index (Model 3).

## References

[1] van den Beld A.W., Kaufman J.-M., Zillikens M.C., Lamberts S.W.J., Egan J.M., van der Lely A.J. (2018). The Physiology of Endocrine Systems with Ageing. Lancet Diabetes Endocrinol..

[2] Chahal H., Drake W. (2007). The Endocrine System and Ageing. J. Pathol..

[3] Bartke A. (2019). Growth Hormone and Aging: Updated Review. World J. Mens. Health.

[4] Luboshitzky R., Shen-Orr Z., Herer P. (2003). Middle-Aged Men Secrete Less Testosterone at Night Than Young Healthy Men. J. Clin. Endocrinol. Metab..

[5] Yiallouris A., Tsioutis C., Agapidaki E., Zafeiri M., Agouridis A.P., Ntourakis D., Johnson E.O. (2019). Adrenal Aging and Its Implications on Stress Responsiveness in Humans. Front. Endocrinol..

[6] Ohlsson C., Labrie F., Barrett-Connor E., Karlsson M.K., Ljunggren Ö., Vandenput L., Mellström D., Tivesten Å. (2010). Low Serum Levels of Dehydroepiandrosterone Sulfate Predict All-Cause and Cardiovascular Mortality in Elderly Swedish Men. J. Clin. Endocrinol. Metab..

[7] Rutkowski K., Sowa P., Rutkowska-Talipska J., Kuryliszyn-Moskal A., Rutkowski R. (2014). Dehydroepiandrosterone (DHEA): Hypes and Hopes. Drugs.

[8] Ceresini G., Morganti S., Rebecchi I., Freddi M., Ceda G.P., Banchini A., Solerte S.B., Ferrari E., Ablondi F., Valenti G. (2000). Evaluation of the Circadian Profiles of Serum Dehydroepiandrosterone (DHEA), Cortisol, and Cortisol/DHEA Molar Ratio after a Single Oral Administration of DHEA in Elderly Subjects. Metabolism.

[9] Anaissie J., DeLay K.J., Wang W., Hatzichristodoulou G., Hellstrom W.J. (2017). Testosterone Deficiency in Adults and Corresponding Treatment Patterns across the Globe. Transl. Androl. Urol..

[10] Dote-Montero M., Amaro-Gahete F.J., De-la-O A., Jurado-Fasoli L., Gutierrez A., Castillo M.J. (2019). Study of the Association of DHEAS, Testosterone and Cortisol with S-Klotho Plasma Levels in Healthy Sedentary Middle-Aged Adults. Exp. Gerontol..

[11] Labrie F., Martel C., Bélanger A., Pelletier G. (2017). Androgens in Women Are Essentially Made from DHEA in Each Peripheral Tissue According to Intracrinology. J. Steroid Biochem. Mol. Biol..

[12] Jones C., Gwenin C. (2021). Cortisol Level Dysregulation and Its Prevalence—Is It Nature’s Alarm Clock?. Physiol. Rep..

[13] Samaras N., Papadopoulou M.-A., Samaras D., Ongaro F. (2014). Off-Label Use of Hormones as an Antiaging Strategy: A Review. Clin. Interv. Aging.

[14] Dote-Montero M., De-la-O A., Jurado-Fasoli L., Ruiz J.R., Castillo M.J., Amaro-Gahete F.J. (2021). The Effects of Three Types of Exercise Training on Steroid Hormones in Physically Inactive Middle-Aged Adults: A Randomized Controlled Trial. Eur. J. Appl. Physiol..

[15] Sasaki N., Fujiwara S., Yamashita H., Ozono R., Teramen K., Kihara Y. (2016). Impact of Sleep on Osteoporosis: Sleep Quality Is Associated with Bone Stiffness Index. Sleep Med..

[16] Piovezan R.D., Abucham J., dos Santos R.V.T., Mello M.T., Tufik S., Poyares D. (2015). The Impact of Sleep on Age-Related Sarcopenia: Possible Connections and Clinical Implications. Ageing Res. Rev..

[17] Beccuti G., Pannain S. (2011). Sleep and Obesity. Curr. Opin. Clin. Nutr. Metab. Care.

[18] Penev P.D. (2012). Update on Energy Homeostasis and Insufficient Sleep. J. Clin. Endocrinol. Metab..

[19] Chaput J.P. (2014). Sleep Patterns, Diet Quality and Energy Balance. Physiol. Behav..

[20] Zuraikat F.M., Makarem N., Redline S., Aggarwal B., Jelic S., St-Onge M.-P. (2020). Sleep Regularity and Cardiometabolic Heath: Is Variability in Sleep Patterns a Risk Factor for Excess Adiposity and Glycemic Dysregulation?. Curr. Diab. Rep..

[21] Matricciani L., Paquet C., Fraysse F., Grobler A., Wang Y., Baur L., Juonala M., Nguyen M.T., Ranganathan S., Burgner D. (2021). Sleep and Cardiometabolic Risk: A Cluster Analysis of Actigraphy-Derived Sleep Profiles in Adults and Children. Sleep.

[22] Besedovsky L., Lange T., Haack M. (2019). The Sleep-Immune Crosstalk in Health and Disease. Physiol. Rev..

[23] Khosro S., Alireza S., Omid A., Forough S. (2011). Night Work and Inflammatory Markers. Indian J. Occup. Environ. Med..

[24] Gabryelska A., Łukasik Z.M., Makowska J.S., Białasiewicz P. (2018). Obstructive Sleep Apnea: From Intermittent Hypoxia to Cardiovascular Complications via Blood Platelets. Front. Neurol..

[25] Pákó J., Kunos L., Mészáros M., Tárnoki D.L., Tárnoki Á.D., Horváth I., Bikov A. (2019). Decreased Levels of Anti-Aging Klotho in Obstructive Sleep Apnea. Rejuvenation Res..

[26] Nakanishi K., Nishida M., Taneike M., Yamamoto R., Adachi H., Moriyama T., Yamauchi-Takihara K. (2019). Implication of Alpha-Klotho as the Predictive Factor of Stress. J. Investig. Med..

[27] Kredlow M.A., Capozzoli M.C., Hearon B.A., Calkins A.W., Otto M.W. (2015). The Effects of Physical Activity on Sleep: A Meta-Analytic Review. J. Behav. Med..

[28] Strand L.B., Laugsand L.E., Wisløff U., Nes B.M., Vatten L., Janszky I. (2013). Insomnia Symptoms and Cardiorespiratory Fitness in Healthy Individuals: The Nord-Trøndelag Health Study (HUNT). Sleep.

[29] Wang T.Y., Wu Y., Wang T., Li Y., Zhang D. (2018). A Prospective Study on the Association of Sleep Duration with Grip Strength among Middle-Aged and Older Chinese. Exp. Gerontol..

[30] Copinschi G., Caufriez A. (2013). Sleep and Hormonal Changes in Aging. Endocrinol. Metab. Clin. N. Am..

[31] Noël S. (2009). Morbidity of Irregular Work Schedules. Rev. Med. Brux..

[32] Van Cauter E., Knutson K.L. (2008). Sleep and the Epidemic of Obesity in Children and Adults. Eur. J. Endocrinol..

[33] Van Cauter E., Spiegel K., Tasali E., Leproult R. (2008). Metabolic Consequences of Sleep and Sleep Loss. Sleep Med..

[34] Cappuccio F.P., D’Elia L., Strazzullo P., Miller M.A. (2010). Sleep Duration and All-Cause Mortality: A Systematic Review and Meta-Analysis of Prospective Studies. Sleep.

[35] Barrett-Connor E., Dam T.-T., Stone K., Harrison S.L., Redline S., Orwoll E. (2008). The Association of Testosterone Levels with Overall Sleep Quality, Sleep Architecture, and Sleep-Disordered Breathing. J. Clin. Endocrinol. Metab..

[36] Patel P., Shiff B., Kohn T.P., Ramasamy R. (2019). Impaired Sleep Is Associated with Low Testosterone in US Adult Males: Results from the National Health and Nutrition Examination Survey. World J. Urol..

[37] Burschtin O., Wang J. (2016). Testosterone Deficiency and Sleep Apnea. Urol. Clin. N. Am..

[38] Redwine L., Hauger R.L., Gillin J.C., Irwin M. (2000). Effects of Sleep and Sleep Deprivation on Interleukin-6, Growth Hormone, Cortisol, and Melatonin Levels in Humans 1. J. Clin. Endocrinol. Metab..

[39] Brandenberger G., Weibel L. (2004). The 24-h Growth Hormone Rhythm in Men: Sleep and Circadian Influences Questioned. J. Sleep Res..

[40] Van Cauter E., Copinschi G. (2000). Interrelationships between Growthhormone and Sleep. Growth Horm. IGF Res..

[41] Asarnow L.D. (2020). Depression and Sleep: What Has the Treatment Research Revealed and Could the HPA Axis Be a Potential Mechanism?. Curr. Opin. Psychol..

[42] van Dalfsen J.H., Markus C.R. (2018). The Influence of Sleep on Human Hypothalamic–Pituitary–Adrenal (HPA) Axis Reactivity: A Systematic Review. Sleep Med. Rev..

[43] Mohammadi H., Rezaei M., Faghihi F., Khazaie H. (2019). Hypothalamic–Pituitary–Gonadal Activity in Paradoxical and Psychophysiological Insomnia. J. Med. Signals Sens..

[44] Mohammadi H., Rezaei M., Sharafkhaneh A., Khazaie H., Ghadami M.R. (2020). Serum Testosterone/Cortisol Ratio in People with Obstructive Sleep Apnea. J. Clin. Lab. Anal..

[45] Zhang W., Piotrowska K., Chavoshan B., Wallace J., Liu P.Y. (2018). Sleep Duration Is Associated With Testis Size in Healthy Young Men. J. Clin. Sleep Med..

[46] Ruge M., Skaaby T., Andersson A.-M., Linneberg A. (2019). Cross-Sectional Analysis of Sleep Hours and Quality with Sex Hormones in Men. Endocr. Connect..

[47] Bani-Issa W., Radwan H., Al Marzooq F., Al Awar S., Al-Shujairi A.M., Samsudin A.R., Khasawneh W., Albluwi N. (2020). Salivary Cortisol, Subjective Stress and Quality of Sleep Among Female Healthcare Professionals. J. Multidiscip. Healthc..

[48] Jurado-Fasoli L., Amaro-Gahete F.J., De-la-O A., Dote-Montero M., Gutiérrez Á., Castillo M.J. (2018). Association between Sleep Quality and Body Composition in Sedentary Middle-Aged Adults. Medicina.

[49] Jurado-Fasoli L., Mochon-Benguigui S., Castillo M.J., Amaro-Gahete F.J. (2020). Association between Sleep Quality and Time with Energy Metabolism in Sedentary Adults. Sci. Rep..

[50] Mochón-Benguigui S., Carneiro-Barrera A., Castillo M.J., Amaro-Gahete F.J. (2020). Is Sleep Associated with the S-Klotho Anti-Aging Protein in Sedentary Middle-Aged Adults? The FIT-AGEING Study. Antioxidants.

[51] Mochón-Benguigui S., Carneiro-Barrera A., Castillo M.J., Amaro-Gahete F.J. (2021). Role of Physical Activity and Fitness on Sleep in Sedentary Middle-Aged Adults: The FIT-AGEING Study. Sci. Rep..

[52] Luboshitzky R., Herer P., Levi M., Shen-Orr Z., Lavie P. (1999). Relationship between Rapid Eye Movement Sleep and Testosterone Secretion in Normal Men. J. Androl..

[53] Van Cauter E. (2000). Age-Related Changes in Slow Wave Sleep and REM Sleep and Relationship With Growth Hormone and Cortisol Levels in Healthy Men. JAMA.

[54] Saaresranta T., Polo O. (2003). Sleep-Disordered Breathing and Hormones. Eur. Respir. J..

[55] Brandenberger G., Gronfier C., Chapotot F., Simon C., Piquard F. (2000). Effect of Sleep Deprivation on Overall 24 h Growth-Hormone Secretion. Lancet.

[56] Nollet M., Wisden W., Franks N.P. (2020). Sleep Deprivation and Stress: A Reciprocal Relationship. Interface Focus.

[57] Kanikowska D., Roszak M., Rutkowski R., Sato M., Sikorska D., Orzechowska Z., Bręborowicz A., Witowski J. (2019). Seasonal Differences in Rhythmicity of Salivary Cortisol in Healthy Adults. J. Appl. Physiol..

[58] Kruisbrink M., Robertson W., Ji C., Miller M.A., Geleijnse J.M., Cappuccio F.P. (2017). Association of Sleep Duration and Quality with Blood Lipids: A Systematic Review and Meta-Analysis of Prospective Studies. BMJ Open.

[59] Song M.J., Kim J.H. (2021). Family Caregivers of People with Dementia Have Poor Sleep Quality: A Nationwide Population-Based Study. Int. J. Environ. Res. Public Health.

[60] Buysse D.J., Hall M.L., Strollo P.J., Kamarck T.W., Owens J., Lee L., Reis S.E., Matthews K.A. (2008). Relationships between the Pittsburgh Sleep Quality Index (PSQI), Epworth Sleepiness Scale (ESS), and Clinical/Polysomnographic Measures in a Community Sample. J. Clin. Sleep Med..

[61] Berger I., Obeid J., Timmons B.W., DeMatteo C. (2017). Exploring Accelerometer Versus Self-Report Sleep Assessment in Youth With Concussion. Glob. Pediatr. Health.

[62] Sadeh A. (2011). The Role and Validity of Actigraphy in Sleep Medicine: An Update. Sleep Med. Rev..

[63] Goelema M.S., Regis M., Haakma R., van den Heuvel E.R., Markopoulos P., Overeem S. (2019). Determinants of Perceived Sleep Quality in Normal Sleepers. Behav. Sleep Med..

[64] Amaro-Gahete F.J., De-la-O A., Jurado-Fasoli L., Espuch-Oliver A., Robles-Gonzalez L., Navarro-Lomas G., de Haro T., Femia P., Castillo M.J., Gutierrez A. (2018). Exercise Training as S-Klotho Protein Stimulator in Sedentary Healthy Adults: Rationale, Design, and Methodology. Contemp. Clin. Trials Commun..

[65] WHO Obesidad y Sobrepeso. https://www.who.int/es/news-room/fact-sheets/detail/obesity-and-overweight.

[66] Buysse D.J., Reynolds C.F., Monk T.H., Berman S.R., Kupfer D.J. (1989). The Pittsburgh Sleep Quality Index: A New Instrument for Psychiatric Practice and Research. Psychiatry Res..

[67] Migueles J.H., Cadenas-Sanchez C., Ekelund U., Delisle Nyström C., Mora-Gonzalez J., Löf M., Labayen I., Ruiz J.R., Ortega F.B. (2017). Accelerometer Data Collection and Processing Criteria to Assess Physical Activity and Other Outcomes: A Systematic Review and Practical Considerations. Sport. Med..

[68] van Hees V.T., Fang Z., Langford J., Assah F., Mohammad A., da Silva I.C.M., Trenell M.I., White T., Wareham N.J., Brage S. (2014). Autocalibration of Accelerometer Data for Free-Living Physical Activity Assessment Using Local Gravity and Temperature: An Evaluation on Four Continents. J. Appl. Physiol..

[69] van Hees V.T., Sabia S., Anderson K.N., Denton S.J., Oliver J., Catt M., Abell J.G., Kivimäki M., Trenell M.I., Singh-Manoux A. (2015). A Novel, Open Access Method to Assess Sleep Duration Using a Wrist-Worn Accelerometer. PLoS ONE.

[70] Shrivastava D., Jung S., Saadat M., Sirohi R., Crewson K. (2014). How to Interpret the Results of a Sleep Study. J. Community Hosp. Intern. Med. Perspect..

[71] Vermeulen A., Verdonck L., Kaufman J.M. (1999). A Critical Evaluation of Simple Methods for the Estimation of Free Testosterone in Serum. J. Clin. Endocrinol. Metab..

[72] Edwards B., O’Driscoll D., Ali A., Jordan A., Trinder J., Malhotra A. (2010). Aging and Sleep: Physiology and Pathophysiology. Semin. Respir. Crit. Care Med..

[73] Carneiro-Barrera A., Amaro-Gahete F.J., Acosta F.M., Ruiz J.R. (2020). Body Composition Impact on Sleep in Young Adults: The Mediating Role of Sedentariness, Physical Activity, and Diet. J. Clin. Med..

[74] Sidhu S., Parikh T., Burman K.D. (2016). Endocrine Changes in Obesity. Perioperative Anesthetic Care of the Obese Patient.

