# Supplementary material for: Sleep and Anabolic/Catabolic Hormonal Profile in Sedentary Middle-Aged Adults: The FIT-AGEING Study
Source: Int J Mol Sci. 2022 Nov 25;23(23):14709. doi: 10.3390/ijms232314709 (PMC9739476; doi:10.3390/ijms232314709)
Supplement: Supplementary file 1 [file ijms-23-14709-s001.zip › ijms-2014699-supplementary.pdf]

SUPPLEMENTARY MATERIAL

Table S1. Association of sleep quantity and quality with DHEAS, free testosterone, somatotropin, and cortisol (Model 0) adjusted by age (Model 1), by fat mass index (Model 2), and by lean mass index (Model 3).

	All			Men			Women		
	β	R^2	*p*	*β*	R^2	*p*	*β*	R^2	*p*
**Global PSQI score**									
DHEAS (μg/dL)									
Model 0	–0.300	0.090	**0.015**	–0.195	0.038	0.301	–0.281	0.079	0.097
Model 1	–0.241	0.165	**0.045**	–0.014	0.258	0.939	–0.231	0.163	0.163
Model 2	–0.289	0.097	**0.020**	–0.195	0.038	0.312	–0.276	0.081	0.109
Model 3	–0.116	0.233	0.350	0.024	0.138	0.912	–0.191	0.158	0.264
Free Testosterone (ng/dL)									
Model 0	–0.249	0.062	**0.044**	–0.144	0.021	0.449	–0.109	0.012	0.527
Model 1	–0.281	0.085	**0.026**	–0.146	0.021	0.482	–0.105	0.012	0.552
Model 2	–0.216	0.125	0.073	–0.137	0.031	0.476	–0.090	0.053	0.602
Model 3	0.055	0.452	0.598	0.144	0.192	0.501	–0.142	0.023	0.436
Somatotropin (ng/mL)									
Model 0	0.081	0.007	0.517	0.122	0.015	0.521	–0.035	0.001	0.840
Model 1	0.079	0.007	0.543	–0.009	0.129	0.965	–0.024	0.005	0.891
Model 2	0.117	0.084	0.339	0.137	0.073	0.467	0.009	0.211	0.952
Model 3	–0.051	0.080	0.706	0.027	0.034	0.907	–0.048	0.003	0.796
Cortisol (μg/dL)									
Model 0	–0.308	0.095	**0.012**	–0.227	0.052	0.227	–0.339	0.115	**0.043**
Model 1	–0.355	0.144	**0.004**	–0.336	0.131	0.092	–0.365	0.138	**0.033**
Model 2	–0.301	0.098	**0.015**	–0.215	0.089	0.252	–0.351	0.130	**0.039**
Model 3	–0.295	0.096	**0.030**	–0.280	0.058	0.228	–0.346	0.116	0.053
**Total sleep time**									
DHEAS (μg/dL)									
Model 0	–0.176	0.031	0.145	0.100	0.010	0.580	–0.150	0.022	0.377
Model 1	–0.200	0.136	0.084	0.109	0.221	0.502	–0.167	0.132	0.303
Model 2	–0.168	0.041	0.166	0.107	0.012	0.565	–0.163	0.031	0.347
Model 3	0.016	0.264	0.886	0.125	0.196	0.450	–0.116	0.138	0.475
Free testosterone (ng/dL)									
Model 0	–0.328	0.108	**0.006**	–0.145	0.021	0.421	0.232	0.054	0.167
Model 1	–0.326	0.109	**0.006**	–0.143	0.033	0.433	0.231	0.055	0.176
Model 2	–0.307	0.175	**0.008**	–0.108	0.090	0.543	0.212	0.075	0.212
Model 3	–0.098	0.442	0.324	–0.124	0.151	0.469	0.228	0.055	0.182
Somatotropin (ng/mL)									
Model 0	0.280	0.078	**0.019**	0.040	0.002	0.825	0.172	0.029	0.310
Model 1	0.277	0.080	**0.021**	0.034	0.083	0.847	0.169	0.032	0.324
Model 2	0.295	0.114	**0.013**	0.032	0.005	0.863	0.118	0.174	0.459
Model 3	0.181	0.139	0.143	0.029	0.037	0.874	0.171	0.030	0.321
Cortisol (μg/dL)									
Model 0	–0.094	0.009	0.438	0.103	0.011	0.570	–0.204	0.042	0.226
Model 1	–0.082	0.039	0.499	0.098	0.063	0.584	–0.199	0.049	0.242
Model 2	–0.086	0.018	0.481	0.134	0.060	0.460	–0.200	0.042	0.246
Model 3	–0.050	0.021	0.703	0.105	0.012	0.567	–0.198	0.045	0.248
**Wake after sleep onset**									
DHEAS (μg/dL)									
Model 0	0.076	0.006	0.533	0.039	0.002	0.830	0.068	0.005	0.688
Model 1	0.083	0.103	0.476	–0.052	0.212	0.756	0.175	0.132	0.302
Model 2	0.060	0.017	0.625	0.032	0.002	0.868	0.073	0.010	0.672
Model 3	0.018	0.264	0.866	–0.038	0.182	0.820	0.090	0.133	0.579
Free Testosterone (ng/dL)									
Model 0	0.062	0.004	0.608	0.104	0.011	0.563	–0.168	0.028	0.320
Model 1	0.061	0.007	0.617	0.086	0.020	0.645	–0.172	0.028	0.337
Model 2	0.021	0.082	0.862	0.028	0.079	0.880	–0.158	0.056	0.351
Model 3	–0.012	0.433	0.896	0.040	0.138	0.820	–0.172	0.033	0.314
Somatotropin (ng/mL)									
Model 0	–0.103	0.011	0.397	0.012	0.000	0.948	–0.137	0.019	0.418
Model 1	–0.101	0.014	0.406	0.070	0.086	0.698	–0.131	0.019	0.465
Model 2	–0.130	0.044	0.285	0.032	0.005	0.869	–0.113	0.173	0.475
Model 3	–0.066	0.115	0.570	0.047	0.039	0.796	–0.139	0.020	0.419

Cortisol (μg/dL)									
Model 0	0.015	0.000	0.899	–0.249	0.062	0.162	0.393	0.154	**0.016**
Model 1	0.011	0.032	0.926	–0.212	0.097	0.239	0.398	0.155	**0.021**
Model 2	0.000	0.011	0.999	–0.334	0.145	0.067	0.391	0.155	**0.018**
Model 3	0.000	0.019	0.999	–0.264	0.069	0.150	0.399	0.165	**0.016**
**Sleep efficiency**									
DHEAS (μg/dL)									
Model 0	–0.106	0.011	0.382	–0.005	0.000	0.978	–0.099	0.010	0.560
Model 1	–0.114	0.110	0.328	0.072	0.215	0.664	–0.184	0.136	0.269
Model 2	–0.090	0.021	0.467	0.005	0.001	0.981	–0.104	0.016	0.545
Model 3	–0.002	0.263	0.985	0.070	0.185	0.677	–0.106	0.136	0.512
Free Testosterone (ng/dL)									
Model 0	–0.152	0.023	0.208	–0.128	0.016	0.477	0.203	0.041	0.229
Model 1	–0.151	0.026	0.215	–0.113	0.025	0.542	0.205	0.041	0.243
Model 2	–0.108	0.093	0.363	–0.055	0.081	0.764	0.191	0.067	0.257
Model 3	–0.020	0.434	0.836	–0.067	0.140	0.701	0.204	0.045	0.232
Somatotropin (ng/mL)									
Model 0	0.156	0.024	0.198	–0.012	0.000	0.947	0.164	0.027	0.332
Model 1	0.154	0.028	0.205	–0.061	0.085	0.734	0.159	0.027	0.368
Model 2	0.188	0.062	0.121	–0.032	0.005	0.869	0.137	0.179	0.387
Model 3	0.092	0.119	0.436	–0.046	0.038	0.800	0.164	0.028	0.338
Cortisol (μg/dL)									
Model 0	–0.020	0.000	0.871	0.228	0.052	0.202	–0.363	0.132	**0.027**
Model 1	–0.015	0.032	0.898	0.195	0.090	0.277	–0.360	0.132	**0.035**
Model 2	–0.002	0.011	0.984	0.309	0.131	0.091	–0.361	0.133	**0.031**
Model 3	0.009	0.019	0.944	0.242	0.058	0.189	–0.365	0.139	**0.028**

β (standardized regression coefficient), R^2, and *p*-value of simple and multiple-regression analysis. Significant *p*-values (< 0.05) are in bold. PSQI: Pittsburgh Sleep Quality Index; DHEAS: dehydroepiandrosterone sulphate.

Table S2. Association of sleep quantity and quality with total testosterone, SHBG, and DHEAS/cortisol, free testosterone/cortisol, total testosterone/cortisol, and somatotropin/cortisol ratios (Model 0) adjusted by age (Model 1), by fat mass index (Model 2), and by lean mass index (Model 3).

	All			Men			Women		
	β	R^2	*p*	*β*	R^2	*p*	*β*	R^2	*p*
**Global PSQI score**									
Total testosterone (ng/dL)									
Model 0	–0.194	0.038	0.118	0.027	0.001	0.888	–0.071	0.005	0.682
Model 1	–0.247	0.100	**0.047**	–0.055	0.046	0.786	–0.085	0.012	0.631
Model 2	–0.160	0.109	0.188	0.038	0.033	0.843	–0.054	0.035	0.756
Model 3	0.124	0.466	0.230	0.275	0.129	0.219	–0.096	0.011	0.600
SHBG (nmol/L)									
Model 0	0.072	0.005	0.564	–0.059	0.003	0.759	–0.010	0.000	0.955
Model 1	0.094	0.015	0.467	–0.166	0.081	0.409	0.017	0.024	0.925
Model 2	0.091	0.027	0.469	–0.054	0.009	0.781	0.028	0.152	0.864
Model 3	–0.054	0.073	0.688	0.022	0.017	0.926	0.012	0.005	0.947
DHEAS/cortisol ratio									
Model 0	–0.171	0.029	0.169	–0.128	0.016	0.502	–0.160	0.026	0.351
Model 1	–0.123	0.082	0.324	–0.002	0.121	0.991	–0.119	0.081	0.485
Model 2	–0.163	0.034	0.197	–0.128	0.016	0.508	–0.150	0.037	0.389
Model 3	–0.050	0.091	0.708	–0.014	0.043	0.950	–0.085	0.081	0.630
Free testosterone/cortisol ratio									
Model 0	–0.192	0.037	0.122	–0.101	0.010	0.597	–0.041	0.002	0.813
Model 1	–0.200	0.038	0.119	–0.012	0.063	0.952	–0.050	0.005	0.778
Model 2	–0.171	0.064	0.169	–0.102	0.011	0.599	–0.024	0.033	0.891
Model 3	0.053	0.291	0.656	0.075	0.074	0.743	–0.074	0.013	0.685
Total testosterone/cortisol ratio									
Model 0	–0.154	0.024	0.218	–0.005	0.000	0.978	0.011	0.000	0.950
Model 1	–0.178	0.036	0.165	0.059	0.028	0.773	–0.011	0.017	0.948
Model 2	–0.130	0.057	0.295	–0.006	0.000	0.976	0.025	0.022	0.885
Model 3	0.124	0.350	0.276	0.210	0.096	0.355	–0.018	0.008	0.923
Somatotropin/cortisol ratio									
Model 0	0.147	0.021	0.240	0.114	0.013	0.549	0.066	0.004	0.701
Model 1	0.147	0.021	0.254	–0.014	0.122	0.943	0.074	0.006	0.679
Model 2	0.186	0.115	0.125	0.129	0.071	0.494	0.116	0.274	0.440
Model 3	0.014	0.096	0.916	0.020	0.031	0.932	0.055	0.006	0.765
**Total sleep time**									
Total testosterone (ng/dL)									
Model 0	–0.320	0.103	**0.007**	–0.017	0.000	0.927	0.222	0.049	0.188
Model 1	–0.311	0.118	**0.009**	–0.018	0.004	0.922	0.226	0.056	0.184
Model 2	–0.301	0.158	**0.009**	0.016	0.056	0.928	0.204	0.064	0.231
Model 3	–0.082	0.460	0.400	0.001	0.086	0.997	0.219	0.050	0.202
SHBG (nmol/L)									
Model 0	0.344	0.119	**0.004**	0.202	0.041	0.259	0.165	0.027	0.329
Model 1	0.334	0.138	**0.005**	0.204	0.048	0.262	0.158	0.044	0.353
Model 2	0.358	0.147	**0.002**	0.209	0.043	0.255	0.109	0.183	0.491
Model 3	0.280	0.145	**0.025**	0.223	0.165	0.192	0.172	0.032	0.318
DHEAS/cortisol ratio									
Model 0	–0.105	0.011	0.388	0.067	0.004	0.713	–0.138	0.019	0.416
Model 1	–0.125	0.086	0.291	0.074	0.138	0.665	–0.152	0.086	0.362
Model 2	–0.099	0.015	0.417	0.068	0.005	0.715	–0.154	0.032	0.372
Model 3	0.024	0.116	0.845	0.082	0.075	0.643	–0.112	0.087	0.502
Free testosterone/cortisol ratio									
Model 0	–0.228	0.052	0.057	–0.022	0.000	0.904	0.217	0.047	0.197
Model 1	–0.232	0.055	0.055	–0.016	0.084	0.929	0.220	0.051	0.197
Model 2	–0.214	0.084	0.073	–0.010	0.008	0.957	0.200	0.061	0.241
Model 3	–0.037	0.282	0.741	–0.007	0.061	0.968	0.211	0.051	0.218
Total testosterone/cortisol ratio									
Model 0	–0.245	0.060	**0.041**	0.015	0.000	0.933	0.199	0.039	0.239
Model 1	–0.244	0.060	**0.044**	0.020	0.061	0.909	0.206	0.060	0.224
Model 2	–0.231	0.087	0.053	0.024	0.004	0.898	0.185	0.048	0.280
Model 3	–0.040	0.324	0.716	0.030	0.060	0.868	0.192	0.044	0.262
Somatotropin/cortisol ratio									
Model 0	0.299	0.090	**0.012**	–0.031	0.001	0.862	0.210	0.044	0.212
Model 1	0.297	0.090	**0.013**	–0.036	0.044	0.843	0.209	0.044	0.221
Model 2	0.318	0.139	**0.007**	–0.033	0.001	0.858	0.150	0.224	0.332

Model 3	0.194	0.159	0.112	–0.042	0.031	0.819	0.207	0.045	0.228
**Wake after sleep onset**									
Total testosterone (ng/dL)									
Model 0	0.054	0.003	0.657	0.048	0.002	0.789	–0.157	0.025	0.354
Model 1	0.051	0.024	0.676	0.063	0.008	0.737	–0.194	0.040	0.277
Model 2	0.016	0.069	0.897	–0.019	0.056	0.917	–0.148	0.045	0.384
Model 3	–0.022	0.455	0.805	–0.004	0.086	0.981	–0.161	0.028	0.350
SHBG (nmol/L)									
Model 0	–0.059	0.004	0.625	0.101	0.010	0.576	–0.119	0.014	0.483
Model 1	–0.056	0.030	0.646	0.089	0.014	0.635	–0.086	0.026	0.629
Model 2	–0.082	0.026	0.505	0.103	0.010	0.591	–0.094	0.180	0.551
Model 3	–0.028	0.078	0.811	0.041	0.117	0.814	–0.116	0.016	0.499
DHEAS/cortisol ratio									
Model 0	0.057	0.003	0.638	0.103	0.011	0.568	–0.113	0.013	0.504
Model 1	0.063	0.075	0.591	0.034	0.133	0.846	–0.046	0.065	0.794
Model 2	0.047	0.008	0.702	0.112	0.012	0.558	–0.108	0.021	0.530
Model 3	0.019	0.116	0.869	0.058	0.072	0.747	–0.098	0.084	0.557
Free testosterone/cortisol ratio									
Model 0	0.084	0.007	0.489	0.136	0.019	0.449	–0.184	0.034	0.275
Model 1	0.085	0.009	0.487	0.083	0.091	0.642	–0.216	0.045	0.226
Model 2	0.056	0.041	0.643	0.122	0.021	0.524	–0.176	0.053	0.301
Model 3	0.024	0.282	0.816	0.095	0.070	0.599	–0.190	0.043	0.267
Total testosterone/cortisol ratio									
Model 0	0.096	0.009	0.427	0.158	0.025	0.380	–0.173	0.030	0.307
Model 1	0.096	0.010	0.433	0.115	0.073	0.527	–0.229	0.066	0.194
Model 2	0.071	0.038	0.560	0.154	0.025	0.419	–0.166	0.042	0.331
Model 3	0.033	0.324	0.747	0.118	0.073	0.513	–0.178	0.039	0.297
Somatotropin/cortisol ratio									
Model 0	–0.127	0.016	0.296	0.088	0.008	0.625	–0.203	0.041	0.229
Model 1	–0.126	0.018	0.303	0.133	0.060	0.466	–0.213	0.042	0.232
Model 2	–0.159	0.063	0.187	0.099	0.009	0.607	–0.176	0.232	0.251
Model 3	–0.088	0.134	0.446	0.123	0.044	0.504	–0.207	0.045	0.227
**Sleep efficiency**									
Total testosterone (ng/dL)									
Model 0	–0.146	0.021	0.229	–0.063	0.004	0.729	0.188	0.035	0.265
Model 1	–0.142	0.042	0.239	–0.075	0.009	0.687	0.217	0.050	0.216
Model 2	–0.105	0.079	0.380	0.003	0.055	0.989	0.178	0.055	0.294
Model 3	–0.009	0.454	0.921	–0.012	0.086	0.945	0.189	0.038	0.269
SHBG (nmol/L)									
Model 0	0.135	0.018	0.266	–0.051	0.003	0.778	0.144	0.021	0.396
Model 1	0.131	0.044	0.277	–0.039	0.008	0.835	0.117	0.033	0.503
Model 2	0.162	0.045	0.184	–0.049	0.003	0.800	0.115	0.184	0.463
Model 3	0.082	0.084	0.496	0.008	0.116	0.964	0.143	0.023	0.406
DHEAS/cortisol ratio									
Model 0	–0.076	0.006	0.531	–0.070	0.005	0.697	0.061	0.004	0.721
Model 1	–0.083	0.078	0.484	–0.011	0.132	0.950	0.002	0.063	0.989
Model 2	–0.066	0.010	0.596	–0.076	0.005	0.690	0.054	0.012	0.752
Model 3	–0.008	0.115	0.948	–0.026	0.069	0.886	0.056	0.078	0.738
Free testosterone/cortisol ratio									
Model 0	–0.142	0.020	0.242	–0.123	0.015	0.495	0.212	0.045	0.208
Model 1	–0.143	0.022	0.242	–0.078	0.090	0.664	0.236	0.055	0.177
Model 2	–0.112	0.050	0.355	–0.107	0.018	0.573	0.203	0.063	0.232
Model 3	–0.035	0.283	0.739	–0.083	0.068	0.647	0.214	0.052	0.209
Total testosterone/cortisol ratio									
Model 0	–0.159	0.025	0.187	–0.139	0.019	0.440	0.196	0.039	0.244
Model 1	–0.159	0.026	0.192	–0.101	0.071	0.574	0.240	0.072	0.167
Model 2	–0.133	0.051	0.275	–0.133	0.020	0.484	0.189	0.050	0.268
Model 3	–0.046	0.325	0.655	–0.100	0.069	0.580	0.198	0.046	0.246
Somatotropin/cortisol ratio									
Model 0	0.178	0.032	0.141	–0.091	0.008	0.614	0.228	0.052	0.174
Model 1	0.177	0.034	0.146	–0.128	0.059	0.480	0.235	0.053	0.179
Model 2	0.216	0.084	0.073	–0.101	0.010	0.597	0.198	0.240	0.195
Model 3	0.110	0.138	0.344	–0.124	0.044	0.499	0.229	0.055	0.178

β (standardized regression coefficient), R^2, and p-value of simple and multiple-regression analysis. Significant p-values (< 0.05) are in bold. PSQI: Pittsburgh Sleep Quality Index; SHBG: sex hormone binding globulin; DHEAS: dehydroepiandrosterone sulphate.
